# Improvement of plan quality in whole-breast radiation following BCS using feasibility DVH by less-experienced planners

**DOI:** 10.1007/s13246-024-01493-y

**Published:** 2024-11-07

**Authors:** Yun Zhang, Yuling Huang, Mingming Luo, Xingxing Yuan, Xiaoping Wang, Changfei Gong

**Affiliations:** Department of Radiation Oncology, Jiangxi Cancer Hospital, Jiangxi Clinical Research Center for Cancer, JXHC Key Laboratory of Tumor Microenvironment and Immunoregulation, Nanchang, Jiangxi 330029 PR China

**Keywords:** Left-sided breast-conserving surgery, VMAT, Dosimetry, Feasibility dose-volume histogram, Optimization

## Abstract

Variability in plan quality of radiotherapy is commonly attributed to the planner’s skill rather than technological parameters. While experienced planners can set reasonable parameters before optimization, less experienced planners face challenges. This study aimed to assess the quality of volumetric-modulated arc therapy (VMAT) in patients with left-sided breast cancer following breast-conserving surgery. Twenty-eight patients requiring whole-breast irradiation were randomly selected for inclusion. Each patient underwent two VMAT treatment plans: one optimized by an experienced planner (VMAT-EXP group) and the other designed by a less experienced planner using feasibility dose-volume histogram (FDVH) parameters from PlanIQ (VMAT-FDVH group). Both plans aimed to deliver a prescription dose of 50 Gy in 25 fractions to the planning target volume (PTV). Dosimetry parameters for the PTV and organs at risk (OARs) were compared between the two groups. Both the VMAT-EXP and VMAT-FDVH groups met the clinical plan goals for PTV and OARs. VMAT-FDVH demonstrated a PTV coverage and homogeneity comparable to those of VMAT-EXP. Compared to VMAT-EXP plans, VMAT-FDVH plans resulted in a significant reduction in the mean ipsilateral lung dose, with an average decrease of 0.9 Gy (8.5 Gy vs. 7.6 Gy, *P* < 0.001). The V5Gy and V20Gy of the ipsilateral lung were also reduced by 3.2% and 1.8%, respectively. Minor differences were observed in the heart, contralateral lung, breast, and liver. Personalized objectives derived from the feasibility DVH tool facilitated the generation of acceptable VMAT plans. Less experienced planners achieved lower doses to the ipsilateral lung while maintaining adequate target coverage and homogeneity. These findings suggest the potential for the effective use of VMAT in in patients with left-sided breast cancer following breast-conserving surgery, especially when guided by feasibility DVH parameters.

## Introduction

Breast cancer, one of the most frequently diagnosed cancer, ranks first in cancer incidence among women globally [1]. Breast-conserving surgery (BCS) is the preferred treatment method for early-stage breast cancer [2, 3]. Following BCS, radiation therapy (RT) has emerged as the standard of care for most patients with early invasive breast cancer [4, 5]. RT not only reduces the risk of local failure by approximately half but has recently played a crucial role in enhancing breast cancer outcomes. The focus has shifted toward improving the quality of life of patients during their long-term survival [6, 7]. Consequently, optimization of radiation therapy plans to minimize doses to normal tissues has become a pressing concern in the field of breast cancer RT. Volumetric-modulated arc therapy (VMAT) has garnered attention in clinical settings because of its ability to significantly reduce radiotherapy time and enhance equipment efficiency [8]. In the context of left-sided BCS, Zhao et al. [9] emphasized the advantages of VMAT over intensity-modulated radiation therapy (IMRT), showing improved homogeneity and conformity in planning target volume (PTV) coverage. However, their findings also indicated higher values of mean dose and V5Gy to the left lung and heart with VMAT, suggesting that the use of two tangential fields in IMRT might be beneficial for both adequate PTV coverage and sparing of organs at risk (OARs). However, other studies have reported different results [10–12]. Virén et al. [10] demonstrated that continuous VMAT achieved optimal coverage but at the expense of a significantly increased dose to the contralateral breast. Haciislamoglu et al. [11] reported poor homogeneity in the PTV and elevated doses to OARs with VMAT compared to IMRT. Conversely, Xie et al. [12] recommended VMAT because of its lower doses and risks to the heart, lungs, and contralateral breast. This divergence in the results underscores the need for consistency in planning quality.

Variability in RT plan quality is commonly attributed to the planner’s skill rather than technological parameters [13]. While experienced planners can set reasonable parameters before optimization, less experienced planners face challenges. Although automatic planning (AP) algorithms have been developed to enhance planning consistency [14], the quality of the AP is influenced by the initial requirements and planner experience. Empowering less experienced planners with a priori estimation of achievable goals based on each patient’s unique anatomy can substantially improve plan quality without extensive time investment [15–16].

PlanIQ™ software from Sun Nuclear in Melbourne, Florida, USA, has been promoted as a tool for objectively assessing treatment plan quality, and was previously employed in comparing various treatment plans [17–22]. Recently integrated into the Pinnacle Evolution system, PlanIQ incorporates a feasibility dose-volume histogram (FDVH) to predict the normal tissue dose, aiding planners in setting initial optimization parameters and enhancing plan quality [23–26]. Based on the assumption of dose fall-off from the prescribed dose at the target boundary, PlanIQ provides optimal OARs sparing for a particular patient. Allowing the planning parameters recommended by PlanIQ to be personalized according to the unique anatomical geometry significantly improves the treatment plan quality without many optimizations. For instance, Perumal et al. [23] utilized VMAT plans with OARs goals derived from the RTOG guidelines and FDVH. The incorporation of the FDVH-suggested clinical goals in an autoplan-based optimization significantly improved the plan quality (OAR sparing) with fewer iterative steps. However, several factors must be considered when employing this method. First, it assumes 100% coverage of the PTV by the prescribed dose with ideal PTV homogeneity, differing from typical clinical situations where 95% of the PTV receives the prescribed dose. Second, the FDVH results are technique-independent and do not consider the dose disparities between IMRT and VMAT plans. For instance, in whole left breast irradiation, VMAT plans showed improved PTV homogeneity but higher Dmean and V5Gy to the left lung and heart than IMRT plans for left-sided BCS patients [10–12]. Third, the FDVH independently approximates the lowest boundary of each OAR’s dose-volume histogram (DVH), overlooking the interplay between multiple OARs that should be considered in practical scenarios.

This study aimed to achieve two objectives: (1) to assess whether the quality of plans by less experienced planners, guided by FDVH, could be enhanced in selected whole-breast cases, and (2) to investigate how FDVH improves plan quality in the context of whole-breast planning. To the best of our knowledge, we are the first group to implement the FDVH in whole-breast radiation following BCS by less-experienced planners.

## Methods and materials

### Patients and target delineation

The study included 28 patients with left-sided breast cancer, clinical stage above T1-2, who underwent whole-breast radiation following BCS in our hospital between January 2022 and December 2022 (median age: 44 years, range 30–57 years). This study was approved by the Medical Ethics Committee of our hospital (2023ky001). The patients were immobilized using a thermoplastic mask combined with a custom mold on the Solo Align Full Body System (Klarity, China). The patient was placed in the supine position with both hands above the head. Computed tomography (CT) images for treatment planning were acquired with 5-mm slice thickness from the mastoid process to 3 cm below the fold of the right breast. The clinical target volume (CTV) was delineated according to established guidelines that included the breast [27]. The PTV was created by expanding each CTV by 5 mm margin in all three dimensions and trimming it to 5 mm under the skin. The mean volume and standard deviation of PTV were 587.37 ± 168.11 cm^3^ (range: 363.36–991.93 cm^3^). The right breast, left/right lungs, liver, spinal cord, and heart were contoured on the planning CT images.

### Treatment planning

For each patient, VMAT-EXP and VMAT-FDVH plans were generated by a planner with more than 10 years of planning experience and less than 5 years of planning experience, respectively, using a 3D treatment planning system (Philips Pinnacle version 16.2). An Elekta Infinity linear accelerator with a 6-MV photon energy beam was used for both the VMAT-EXP and VMAT-FDVH plans. All plans with the same two partial arcs starting from 300° to 120°, with a control point every 4°, were optimized using the SmartArt optimization type, and dose calculations were performed using the Collapsed Cone Convolution (CCC) algorithm with a 3 mm x 3 mm x 3 mm calculation grid, and the beam information and parameter information of the planning are the same. All plans had to meet the planning goals (Table 1). To eliminate the influence of other factors, the contours of the auxiliary structures, as well as the initial optimization parameters of PTV, and auxiliary structures were set by loading a predefined technical script prior to planning optimization. The optimization parameters of the OARs, including the heart, left lung, right lung, right breast, and liver, were set based on experience for VMAT-EXP and PlanIQ feasibility DVH for VMAT-FDVH. The prescribed dose was 50 Gy in 25 fractions and the prescription percentage for all plans were normalized to 95% at the mean dose of PTV.

**Table 1:** Planning goal for breast cancer

Structures	Planning aim
PTV	V_47.5 Gy_ ≥98%, V_50_Gy ≥ 95%, V_55_Gy ≤ 0.5%
Heart	mean dose ≤ 6 Gy
Ipsilateral lung	V_5_Gy ≤ 45%, V_20_Gy ≤ 20%, and mean dose < 12 Gy
Contralateral lung	mean dose ≤ 3 Gy
Contralateral breast	mean dose ≤ 3 Gy
Liver	mean dose ≤ 3 Gy
Spinal cord	D_max_ ≤20 Gy

### FDVH in Plan-IQ

**Fig. 1:**
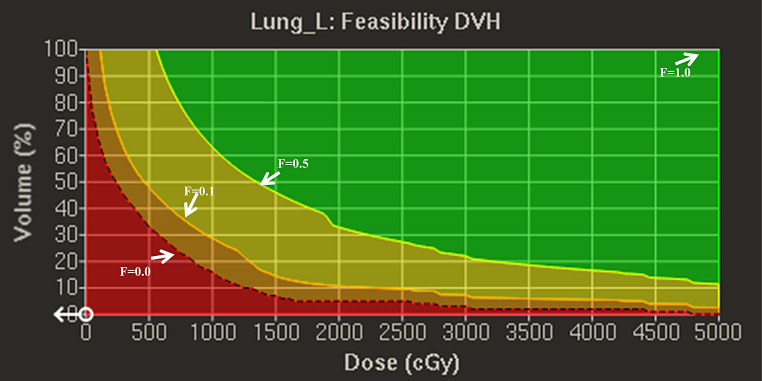
The FDVH for left lung one of a patient, red: impossible region, orange: difficult region, yellow: challenging region, green: probable region

Recently, PlanIQ Feasibility software (SunNuclear, Mel-bourne, FL, USA) was integrated into the recently released Pinnacle Evolution system (Philips Pinnacle version 16.2), which incorporates a FDVH tool to predict the normal tissue dose, aiding planners in setting initial optimization parameters and enhancing plan quality. Based on the assumption of dose fall-off from the prescribed dose at the target boundary, FDVH provides optimal OARs sparing for a particular patient and allowing the recommended planning parameters to be personalized according to the unique anatomical geometry. Figure 1 shows that the FDVH quantitative evaluation tool implemented in PlanIQ identifies four DVH regions: impossible, difficult, challenging, and probable. If the DVH of OAR was in impossible region, it means that the coverage of PTV was less than 100%. The probable region is easy to achieve, and even if there is no OAR target limit, the dose will be within this region. Feasibility is a possibility, expressed as an “F” value, and can be any value between 0 and 1. F = 0 means that the probability of obtaining a plan to the left of the red baseline is 0 (while maintaining 100% coverage of the target area), and F = 1 means that all available plans will exceed the DVH distribution at F = 1. F = 0.00 is the dividing line between impossible and difficult regions, and F = 0.10 is the dividing line between difficult and challenging regions. It’s worth noting that it is inappropriate to place all estimated OARs objectives in a difficult region, and a priori estimated objectives should be selected from the difficult to probable regions based on the priorities of the OARs. For the left lung, the objectives of V5Gy are set by the F = 0.00, V20Gy by the F = 0.10–0.15, and mean dose by the F = 0.06. The initial setting of the mean heart dose was the value provided by F = 0.00. For the contralateral lung and breast, we initially estimated the objectives of the mean dose by F = 0.03. However, the results deviated from the objectives and the coverage of the PTV did not meet the requirements; therefore, we adjusted the estimated objectives by F = 0.03–0.06 region. The estimated objective was evaluated by F = 0.03 in the mean liver dose. Because the spinal cord is not the focus of this study and is farther from the PTV, it can easily meet the clinical requirements, and its adjustment has less impact on the dose distribution in the PTV as well as other OARs; therefore, we did not set it based on the feasibility of DVH. The effect of the average dose to the OARs on the feasibility of DVH with different F values is shown in Table 2.

### Plan evaluation and statistical tools

To evaluate the quality of the plans for different methods, the quantitative metrics of Dmean, D2%, D98% (dose received by 2% and 98% of PTV, near maximum dose and minimum dose), V47.5 Gy, dose homogeneity index (HI), and conformity number (CN) for PTV were calculated and compared. The HI [28] and CN [29] were obtained as:

1 $$\:HI\:=\:(D2\%\--D98\%)/D50\%$$

2 $$\:CN\:=\:(VPTV_{50}/VPTV)*(VPTV_{50}/V_{50})$$

where, VPTV is the target volume, V_50_ is the volume of the prescribed isodose value and VPTV_50_ is the volume of the target covered by the prescribed isodose value. The dosimetry parameters of the OARs included the mean doses to the left lung, heart, right lung, right breast, and liver. The volumes of the left lung receiving 5 Gy (V5 Gy) and 20 Gy (V20 Gy) were also evaluated. The normal tissue complication probability (NTCP) for radiation-induced pneumonitis was calculated for the lung using the Lyman-Kutcher-Berman model using the following values: D50 = 30.80 Gy, *n* = 0.98 and m = 0.37 [30]. The NTCP for radiation-induced mortality for the heart was calculated using the relative seriality model using the following values: D50 = 52.4 Gy, s = 1.0 and γ = 1.3 [31]. Wilcoxon’s signed-rank test was performed to calculate the statistical significance with 95% confidence interval to compare different techniques. A *p*-value ≤ 0.05 was considered statistically significant.

## Results

**Fig. 2:**
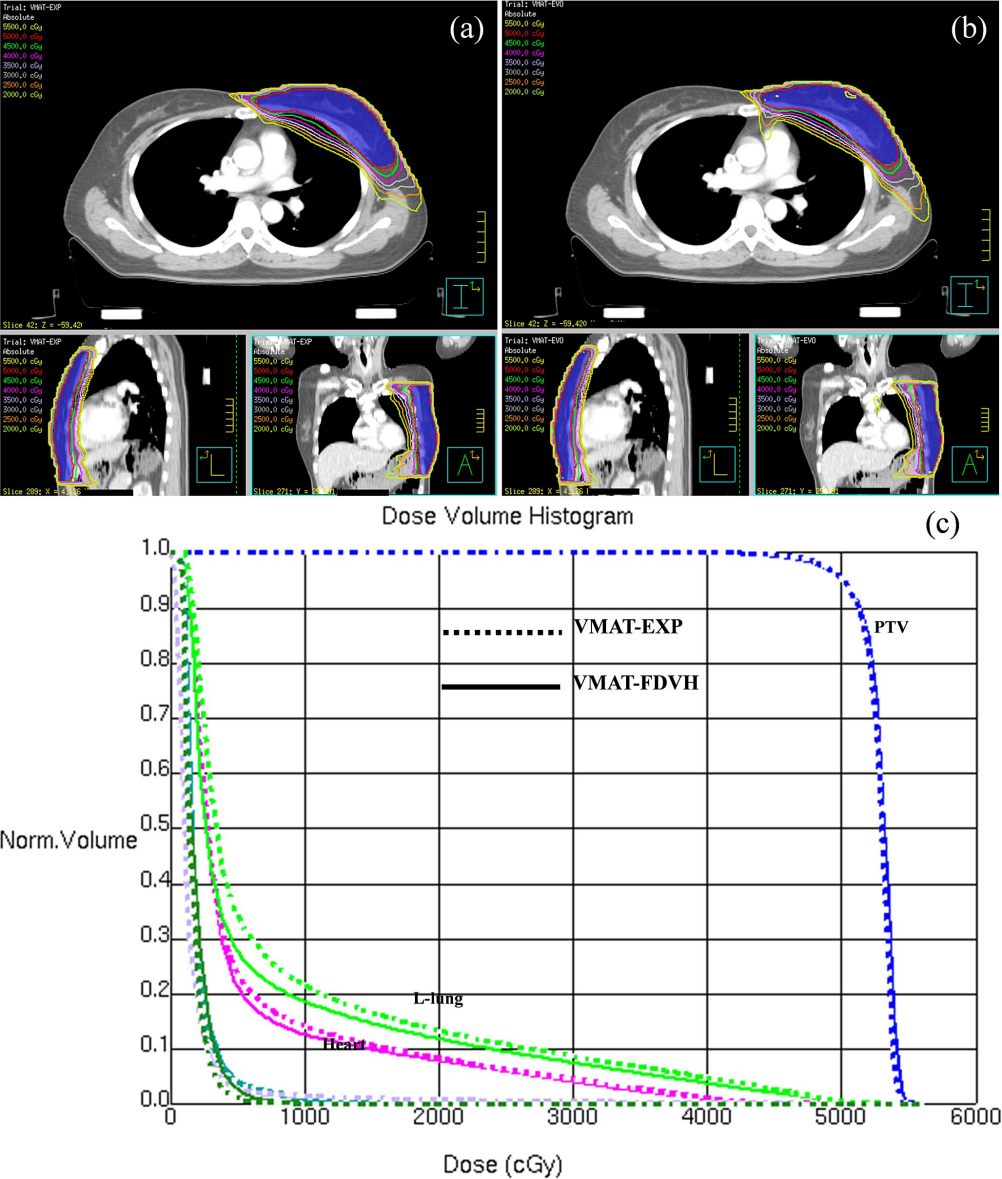
A direct comparison of dose distribution and DVH between VMAT-EXP and VMAT-FDVH. (**a**) VMAT-EXP (**b**) VMAT-FDVH (**c**) DVH, blue: PTV, green: left lung, purple: heart, teal: right breast, forest: right lung, lavender: liver

The transverse, coronal, and sagittal dose distributions of one patient are shown in Fig. 2a and b. The isodose lines were conformal and constricted to the target in both VMAT-EXP and VMAT-FDVH, and the hotspots in the VMAT-FDVH plans were slightly higher than those in the VMAT-EXP plans. For better illustration, Fig. 2c shows the dosimetry differences of the target volume and OARs in a DVH for this patient.

Additional dosimetry parameters of the PTV and OARs of the two VMAT plans for all patients are listed in Tables 2 and 3. The clinical plan goals for the PTV and OARs were achieved with both VMAT-EXP and VMAT-FDVH. No significant differences were found between the VMAT-EXP and VMAT-FDVH plans for the PTV. However, the VMAT-FDVH plans demonstrated a reduction in V5Gy, V20Gy, and Dmean of the left lung; V5Gy by 3.2%, V20Gy by 1.8% and the mean dose by 0.9 Gy. Additionally, VMAT-FDVH plans exhibited a lower dose in the right lung (1.6 ± 0.2 Gy vs. 1.7 ± 0.3 Gy) and breast (1.7 ± 0.3 Gy vs. 2.0 ± 0.3 Gy). For the heart and liver, the differences in the mean doses did not reach statistical significance. NTCP of the left lung, heart, and right lung were reduced by an average of 0.31%, 0.01%, and 0.05%, respectively, in VMAT-FDVH plans compared to that of VMAT-EXP.

**Table 2:** Comparison of PTV and OARs format-EXP and VMAT-FDVH

Structures	parameters	VMAT-EXP	VMAT-FDVH	Difference	*p*
PTV	V_47.5 Gy_ (%)	98.7 ± 0.2	98.6 ± 0.2	-0.1	0.009
	D_2%_(Gy)	54.2 ± 0.3	54.2 ± 0.3	0.0	0.119
	D_98%_(Gy)	48.4 ± 0.2	48.4 ± 0.2	0.1	0.084
	D_mean_(Gy)	52.5 ± 0.2	52.6 ± 0.2	0.1	0.002
	HI	0.11 ± 0.01	0.11 ± 0.01	0.0	0.232
	CN	0.92 ± 0.01	0.93 ± 0.01	0.1	0.523
L-lung	V_5_ (%)	33.4 ± 4.6	30.2 ± 4.9	-3.2	< 0.001
	V_20_ (%)	13.8 ± 2.6	11.9 ± 2.6	-1.8	< 0.001
	D_mean_(Gy)	8.5 ± 1.1	7.6 ± 1.1	-0.9	< 0.001
	NTCP	1.86 ± 0.41	1.55 ± 034		
Heart	D_mean_	4.0 ± 0.9	3.9 ± 1.0	-0.1	0.376
	NTCP	0.18 ± 0.20	0.13 ± 0.15		
R-lung	D_mean_	1.6 ± 0.2	1.7 ± 0.3	0.1	0.001
	NTCP	0.44 ± 0.0.2	0.45 ± 0.03		
R-breast	D_mean_	1.7 ± 0.3	2.0 ± 0.3	0.3	< 0.001
Liver	D_mean_	1.2 ± 0.5	1.2 ± 0.4	0.0	0.329

**Table 3:** The dose of OARs on feasibility DVH when setting F as a different value

OARs	parameters	F = 0.00	F = 0.03	F = 0.06	F = 0.10
L-lung	V_5_ (%)	32.4 ± 5.3	37.8 ± 4.9	41.9 ± 4.6	47.0 ± 4.3
	V_20_ (%)	2.7 ± 1.2	4.9 ± 1.2	6.6 ± 1.3	8.8 ± 1.2
	D_mean_ (Gy)	5.8 ± 0.7	6.7 ± 0.7	7.5 ± 0.7	8.5 ± 0.7
Heart	D_mean_ (Gy)	4.3 ± 1.2	5.1 ± 1.1	5.9 ± 1.1	7.0 ± 1.1
R- lung	D_mean_ (Gy)	0.2 ± 0.1	1.2 ± 0.1	2.2 ± 0.1	3.4 ± 0.1
R-breast	D_mean_ (Gy)	0.3 ± 0.1	1.3 ± 0.1	2.2 ± 0.1	3.4 ± 0.2
Liver	D_mean_ (Gy)	0.5 ± 0.4	1.5 ± 0.4	2.5 ± 0.4	3.6 ± 0.8

## Discussion

In this study, 28 patients who received left-sided whole-breast RT (WBRT) with VMAT were replanned by a less experienced planner using the FDVH of PlanIQ at a difficulty level. The resulting plans were compared to those designed by an experienced planner. For the PTV, there were no differences in homogeneity, conformality, or coverage between the two plans, including the maximum and minimum doses. However, plan optimization based on the FDVH significantly reduced the irradiation dose to the ipsilateral lung, both on V5Gy, V20Gy, and mean doses, which could potentially lead to an improvement in the patient toxicity burden. In addition, the results of the optimized FDVH were in the difficult region for almost all parameters, except for V20Gy in the left lung. Surprisingly, the mean heart dose and V5Gy of the left lung reached an impossible level, which was better than the original goal. Essentially, the experience of the VMAT-FDVH planner was less than five years and the quality of plans without FDVH assistance was typically lower than that of the experienced planner. As demonstrated by Wang et al. [13], the plan quality of IMRT in left-sided breast cancer patients varied considerably based on planning experiences. The plans created by beginner planners exhibited lower CN of the PTV and higher doses for most OARs compared to that by junior and senior planners.

Overall, the clinical planning goals for PTV and OARs were achieved in both VMAT-EXP and VMAT-FDVH, outperforming published literature [9–12]. The CN and HI of PTV were 0.92 and 0.11, respectively, in this study, and compare favorably with values in other studies: 0.728 ± 0.036 and 0.118 ± 0.018 in a study by Zhao [9], 0.50 ± 0.03 and 0.10 ± 0.01 in a study by Virén [10], and 0.74 ± 0.04 and 0.18 ± 0.02 in a study by Haciislamoglu [11], and 0.8 ± 0.1 in a study by Xie [12]. The mean dose in heart, ipsilateral lung, contralateral lung, and breast were 8.5 ± 1.1 Gy, 4.0 ± 0.9 Gy, 1.7 ± 0.2 Gy, and 2.0 ± 0.3 Gy in our study, respectively, and are comparable to those reported by Zhao 5.0 ± 2.0 Gy, 3.3 ± 1.3 Gy, 0.3 ± 0.1 Gy and 0.9 ± 0.3 Gy [9], Virén 8.7 ± 1.7 Gy, 5.5 ± 2.9 Gy, 1.6 ± 0.7 Gy and 2.6 ± 1.2 Gy [10], and Haciislamoglu 11.08 ± 2.7 Gy, 9.24 ± 2.12 Gy, 2.56 ± 0.73 Gy and 3.03 ± 0.61 Gy 14.0 ± 3.4 [11]. The quality of plans based on PlanIQ FDVH also improved, which is consistent with previous research on the use of PlanIQ in RT planning design [19, 21, 23–26]. Fried et al. [19] used the PlanIQ FDVH in head and neck treatment planning, which resulted in a dose reduction to the larynx and contralateral parotids. Sasaki et al. [21] showed that the improvement in prostate IMRT and VMAT treatment plan quality was influenced more by the dose reduction in the OARs than by the target coverage. Perumal et al. [23] concluded that AP PlanIQ plans were significantly better in terms of OAR sparing than AP RTOG plans without compromising target coverage. Xia et al. [25] reported that better lung sparing was achieved using personalized plan parameter settings based on the FDVH for lung cancer with VMAT. Chen et al. [26] demonstrated that an automated planning strategy generated using plan-IQ FDVH for IMRT after a modified radical mastectomy decreased the PTV score while improving the OAR score.

In WBRT for patients with breast cancer, it is not difficult to design a plan to achieve clinical goals; however, the planner should reduce the dose to OARs, especially to the lungs and heart, because of the high quality of long-term survival in these patients [6, 32]. Therefore, it is a challenge for planners to further reduce the dose to OARs while ensuring that the PTV is adequately irradiated. In the optimization process of traditional WBRT, the planner usually manually adds dosimetry constraints based on the relative positions of the PTV and OARs, considering the characteristics of different technologies. During this iterative optimization, planners modify parameters and experiment with various dosimetry constraints based on the optimized results, aiming to develop a treatment plan that meets the clinical requirements. This process is often time-consuming and relies on the skill of the planner. In addition, if further reduction in normal tissue dose is required, the planner must continually experiment with new dose constraints, adding to their workload. PlanIQ does not always improve the planner’s skill but provides a clinically feasible estimate and a template for optimization objectives. Using the feasibility tool a priori estimation of the most feasible DVH for OARs can be generated to initialize the optimization goals. The advantages of this method include its simplicity and minimal commissioning effort. The dose coverage of PTV is not 100%, and no individual OAR objective function can be further improved without sacrificing at least one other. Therefore, in the designs of the WBRT plan based on the FDVH, the heart, lung, and contralateral breast were considered the most important OARs, because of the risks of major coronary events (the rates of which increased linearly with the mean heart dose) [6], pulmonary toxicity [33], and risk of second primary cancer in the contralateral breast [34]. Therefore, the initial setting of the mean heart dose was the value provided by F = 0.00. For the ipsilateral lung, it is crucial to control the low-dose region in the VMAT plan because it will potentially enhance the estimated risk of secondary tumors and radiation-induced pulmonary fibrosis and pneumonitis [35]. Therefore, we estimated the objectives of V5Gy in the impossible region, V20Gy in the challenging region, and mean dose in the difficult region. For the contralateral lungs and chest, estimating the objective in the range of 0.03 to 0.06 is more appropriate to avoid over-estimating the target coverage.

The results of this study indicate that the doses to the primary OARs in VMAT plans using FDVH for WBRT in left-sided breast cancer, were lower than those achieved by experienced treatment planners. We believe that the use of FDVH allows for quality assurance of the clinical treatment plan. However, this study has several limitations. First, we evaluated only the VMAT plan for patients undergoing WBRT. Other patients including those undergoing postmastectomy RT or with a PTV including the whole breast and supraclavicular nodes, were not included. Second, CT scans were performed during free-breathing, and additional studies are needed to determine whether the dose to the heart and lungs can be further reduced in patients using deep inspiration breath-holding techniques. Additionally, we did not combine FDVH with auto-planning, which could further reduce the dependence of plan quality on planning skills. Combining FDVH with auto-planning, is expected to result in higher quality plans more efficiently, and further research is needed in this area in the future.

## Conclusions

Here, we evaluated the quality of the VMAT plan using personalized plan parameters provided by the PlanIQ FDVH in patients with left-sided BCS. Using personalized objectives generated by the FDVH tool, less experienced planners achieved lower doses to the ipsilateral lung, while ensuring adequate target coverage and homogeneity. Although the doses to the contralateral lung and breast were increased, they were negligible.

## Data Availability

The data that support the findings of this study are not openly available due to reasons of sensitivity and are available from the corresponding author upon reasonable request.

## Abbreviations

AP: Automatic planning
BCS: Breast-conserving surgery
CCC: Collapsed Cone Convolution
CN: Conformity number
CT: Computed tomography
CTV: Clinical target volume
DVH: Dose-volume histogram
FDVH: Feasibility dose-volume histogram
HI: Homogeneity index
IMRT: Intensity-modulated radiation therapy
Mus: Monitor units
NTCP: Normal tissue complication probability
OAR: Organs at risk
PTV: Planning target volume
RT: Radiation therapy
VMAT: Volumetric-modulated arc therapy
